# Alpha-Synuclein, cyclooxygenase-2 and prostaglandins-EP2 receptors as neuroinflammatory biomarkers of autism spectrum disorders: Use of combined ROC curves to increase their diagnostic values

**DOI:** 10.1186/s12944-021-01578-7

**Published:** 2021-11-06

**Authors:** Afaf El-Ansary, Manan Alhakbany, Abeer Aldbass, Hanan Qasem, Sarah Al-Mazidi, Ramesa Shafi Bhat, Laila Al-Ayadhi

**Affiliations:** 1 Central Laboratory, Female Center for Medical Studies and Scientific Section, King Saud University, P. O Box 22452, Riyadh, KSA 11495 Saudi Arabia; 2 Autism Research and Treatment Center, Riyadh, Saudi Arabia; 3 Department of Physiology, Faculty of Medicine, King Saud University, Riyadh, Saudi Arabia; 4 Biochemistry Department, College of Science, King Saud University, Riyadh, Saudi Arabia; 5 Department of Physiology, College of Medicine, Al-Imam Mohammed Bin Saud Islamic University, Riyadh, Saudi Arabia

**Keywords:** Autism, Neuroinflammation, α-Synuclein, Cyclooxygenase-2, Prostaglandin-EP2 receptors

## Abstract

**Background:**

Autism spectrum disorder (ASD) is a neurodevelopmental disorder characterized by impairments in social interaction and restricted and repetitive behaviors. Neuroinflammation and abnormal lipid mediators have been identified in multiple investigations as an acknowledged etiological mechanism of ASD that can be targeted for therapeutic intervention.

**Methods:**

In this study, multiple regression and combined receiver operating characteristic (ROC) curve analyses were used to determine the relationship between the neuroinflammatory marker α-synuclein and lipid mediator markers related to inflammation induction, such as cyclooxygenase-2 and prostaglandin-EP2 receptors, in the etiology of ASD. Additionally, the study aimed to determine the linear combination that maximizes the partial area under ROC curves for a set of markers. Forty children with ASD and 40 age- and sex-matched controls were enrolled in the study. Using ELISA, the levels of α-synuclein, cyclo-oxygenase-2, and prostaglandin-EP2 receptors were measured in the plasma of both groups. Statistical analyses using ROC curves and multiple and logistic regression models were performed.

**Results:**

A remarkable increase in the area under the curve was observed using combined ROC curve analyses. Moreover, higher specificity and sensitivity of the combined markers were reported.

**Conclusions:**

The present study indicates that measurement of the predictive value of selected biomarkers related to neuroinflammation and lipid metabolism in children with ASD using a ROC curve analysis should lead to a better understanding of the etiological mechanism of ASD and its link with metabolism. This information may facilitate early diagnosis and intervention.

## Background

Autism spectrum disorder (ASD) is a group of neurodevelopmental disorders. It is characterized by impaired communication skills, deficits in social interaction, and restricted and stereotypic behaviors [1, 2]. Accumulating evidence from rodent models and clinical observations of individuals with ASD suggests that neuroinflammation may contribute considerably to ASD. Some of the crucial features of neuroinflammation are microglial activation with subsequent morphological alterations, increased expression of microglial receptors, and production of neurotrophic and neurotoxic factors [3, 4].

Abruzzo et al. [5] highlighted the benefit of using receiver operating characteristic (ROC) curves as an excellent statistical tool for identifying adequately sensitive biomarkers and specific for early ASD diagnosis. Although their utility in the prediction, risk evaluation, and assessment of therapeutic interventions still requires further studies, ROC curves emphasize the most statistically significant differences between patients and controls. The area under the curve (AUC) provides a valuable measure to assess the predictive value of biomarkers. The area under the curve (AUC) provides a useful measure to assess the predictive value of biomarkers. An AUC value near 1.00 describes an excellent predictive marker, while a curve near the diagonal (AUC = 0.5) has no diagnostic value. An AUC value close to 1.00 is always accompanied by acceptable values of specificity and sensitivity of the biomarker [6]. When studying prospective ASD biomarkers, high sensitivity indicates that ASD will be identified in most patients; in contrast, high specificity means that healthy individuals will rarely be positive for the measured variable. Moreover, ROC curve analyses combining two distinct markers usually increased their specificity [7], suggesting that combining a panel of variables instead of a single variable may be of great value as a diagnostic tool.

As presynaptic proteins, the synuclein protein family is localized to the synaptic terminals of the neocortex, striatum, hippocampus, cerebellum, and cerebellum [8]. The proteins α-synuclein (α-Syn) and β-synuclein (β-Syn) are part of this family, and both have a distinctive consensus sequence and are structurally homologous [8]. Alpha-synuclein plays a critical role in synaptic functions, including synaptic pool preservation, vesicular stabilization, synaptic plasticity, and regulation of dopamine synthesis and release [9]. In relation to the modification of plasma membrane composition and autism, the levels of autoantibodies against ganglioside M1 (GM1), the most abundant ganglioside in neural membranes, are elevated in individuals with autism, and their levels exhibit significant positive correlations with the degree of the severity of the disorder [10]. In a recent study, GM1 administration increased the α-syn clearance from the brain and decreased apoptosis in Alzheimer’s disease models. Thus, elevated levels of GM1 autoantibodies in patients with autism might be related to abnormal brain α-syn clearance, as suggested by the etiological mechanism of autism [10, 11]. It is well documented that ASD are associated with abnormalities in lipid mediators, membrane-associated proteins, and signal transduction [12, 13]. Most recently, in 2021, Man et al. [14] provided proof that changes in the lipid composition of the plasma membrane are usually associated with neurological disorders and alterations in the binding modes of α-Syn. This study might explain how lipid composition controls the interaction of α-Syn with the plasma membrane and triggers its functional and pathological behaviors. Various reports have shown that the clearance of *α*-syn outside the brain is an important mechanism keeping normal levels in the healthy CNS [15, 16]. Patients with ASD have brain anomalies that may affect the blood-brain and/or blood-CSF barriers. Therefore, abnormal *α*-syn clearance to the CSF and blood has been suggested as another etiological mechanism [17, 18]. Moreover, irregularities in α-Syn clearance to the CSF and blood are a probable cause of diminished plasma levels of α-Syn in individuals with ASD. Sriwimol et al. [19] reported that ASD is associated with lower plasma α-Syn levels due to impairments in brain function and development.

Cyclooxygenase (COX) is the rate-limiting enzyme in the synthesis of lipid mediators, such as prostaglandin, prostacyclin, and thromboxane. Two isoforms have been identified: COX-1 and COX-2 [20, 21]. COX-1 is constitutively expressed in several tissues to sustain the homeostasis of prostanoids necessary for many physiological functions. Alternatively, COX-2 is usually present at low levels under normal conditions but is quickly induced by various stimuli to initiate proinflammatory processes that induce and sustain a pathological condition [22]. As a major COX-2 product in the brain, PGE2 has been commonly supposed to promote neuronal inflammation in many neurological disorders. PGE2 binds to and activates four G protein-coupled receptors (GPCRs), EP1, EP2, EP3, and EP4 [23]. These receptors are transmembrane receptors; however they could shed to the plasma through certain proteolytic enzymes known as sheddases. These enzymes, proteolytically cleave the ectodomain (extracellular domain) of hundreds of transmembrane receptors from the cell surface, allowing them to transport in soluble form [24]. Recently, it was found that ADAM10 and ADAM17 as two proteolytic enzymes are highly expressed in the brain as well as the intestine of ASD patients [25]. ADAM10 and ADAM17 regulate synaptic functions, neuroinflammation and early brain development, in addition to intestinal inflammation, intestinal barrier permeability and inflammatory responses leading to leaky gut and gastrointestinal problems as co-morbidity related to the gut-brain axis pathology of ASD [25].

Recently, Sethi et al. [26] recorded that targeting the activated COX2/PGE2 signaling as neuroinflammatory pathway using COX2 inhibitors represent a novel strategy to prevent and treat neuropsychiatric illness in ASD.

In an attempt to understand how α-Syn, PGE2-EP2, and COX-2 contribute to the etiopathology of ASD, COX-2 and PGE2-EP2 were shown to regulate microglial activation-associated neurotoxicity induced by α-Syn aggregation. α-Syn aggregation is toxic to neurons. Jin et al. [27] suggested that the mechanisms by which PGE-EP2^−/−^ knockout mice became more resistant to neurotoxicity were at least somewhat attributed to their greater capability to either prevent the production of or improve clearance of aggregated α-Syn by microglia.

A main achievement in the field of biomarkers is the possibility of measuring neurofilaments in blood as marker of neuronal damage in a wide range of neurological disorders [28, 29].

This information motivated us to use independent and combined ROC curves to measure the diagnostic value of α-Syn, PGE-EP2, and COX-2 as a panel of blood plasma markers and to understand their integrated role in the etiology of ASD.

## Methods

### Participants

The study protocol was accepted by the ethics committee at the College of Medicine, King Saud University, according to the most recent revision to the Declaration of Helsinki (Edinburgh 2000). Two groups of participants were enrolled in the study and comprised 40 male moderate ASD patients and 40 age- and sex-matched healthy controls. Informed consent was obtained from all participants and their parents. Both groups were recruited through the Autism Research & Treatment (ART) Center clinic. The ASD diagnosis was confirmed in all subjects using the Autism Diagnostic Interview-Revised (ADI-R); the Autism Diagnostic Observation Schedule (ADOS); and the Developmental, Dimensional, and Diagnostic Interview (3DI) protocols. The children with ASD were 2–12 years old. All were simplex cases (i.e., the family has one affected individual). All were negative for Fragile X Syndrome gene mutations. The control group was recruited from the pediatric clinic at King Saud Medical City, whose ages ranged from 2 to 13 years. Exclusion criteria included dysmorphic features, Fragile X Syndrome, or other neurological disorders, such as seizures, bipolar disorder, or any known medical conditions. All participants were screened for a history of physical illness by a parental interview. All patients and controls included in the study were on similar, but not identical, diets, and none were on a special gluten-restricted diet.

### Blood sampling

After an overnight fast, blood samples were withdrawn from participants by a skilled technician into 3 mL blood collection tubes containing EDTA. Immediately after collection, blood was centrifuged at 4 °C at 3000 x g for 20 min. The plasma was separated, distributed into three 0.5 mL aliquots (to avoid several freeze-thaw cycles) and stored at − 80 °C until use.

### Ethical approval and consent

This work was officially accepted by the ethics committee of King Khalid Hospital, King Saud University (Approval number: 11/2890/IRB). Written informed consent was obtained from the parents of all participants according to the guidelines of the ethics committee.

### Biochemical assays

#### Cyclooxygenase-2 (COX-2)

COX-2 levels were determined using a quantitative sandwich enzyme-linked immunosorbent assay (ELISA) kit from CUSABIO (8400 Baltimore Avenue, Room 332 College Park, MD 20740). The measurement was performed according to the manufacturer’s provided information and had a minimum detectable dose of 0.31 ng/ml.

#### Prostaglandin (PGE2-EP2)

- PGE2-EP2 levels were determined using an ELISA kit, a product of USCN Life Science (Wuhan, China). It is a sandwich ELISA for the quantitative assay of EP2 in human biological fluids, tissue homogenates and cell lysate, Cataloug No.
  SEA247Hu, detection limit of the kit is 78–5000 pg/ml with minimal detectable concentration of PGE2-EP2 is < 33 pg/ml. http://www.cloud-clone.com/manual/ELISA-Kit-for-Prostaglandin-E-Receptor-2%2D%2DEP2%2D%2DSEA247Hu.pdf

### α-Synuclein

The level of α-Syn in plasma was measured using a sandwich ELISA kit according to the manufacturer’s instructions (IBL- America catalog number 27740). The plasma samples were diluted 10 times with sample buffer provided with the kit to obtain measurable concentrations.

### Statistical analysis

In this study, IBM SPSS software, version 16 (IBM Inc., Armonk, USA) was used to analyze the data. The results are presented as the means ± SD, and ANOVA or independent Fisher’s t-test was used for statistical evaluations, with *P* ≤ 0.05 considered a significant difference. Multiple regression analysis was used to determine the correlation between each of the measured markers as a dependent variable and the other two markers as independent or predictor variables. In multiple regression analyses, R^2^ defines the percentage of change in the dependent variable explained by the change in the predictor variables together. An R^2^ of 1.00 shows that 100% of the changes in the dependent variable are directly related to the independent variables. In contrast, an R^2^ of 0.0 shows the absence of variation in the dependent variable due to the independent or predictor variables. The positive or negative directions in which an independent variable contributes to the change in the dependent variable relative to the other independent variables are usually shown by the ß coefficient values. R^2^ and ß coefficients were sufficient to interpret the multiple regression data. For the combined ROC curves, odds ratios (ORs) obtained from logistic regression analyses describe associations of biomarkers with the clinical status. ROC curves were constructed for each logistic regression model. The area under the curve (ROC-AUC) was compared between each marker and marker combination using a nonparametric method [30]. Likewise, the predictiveness curves, as a complement to ROC curves of the three measured parameters, were drawn using a Biostat 16 computer program and the x-axis represents percentile rank of the biomarker, the y-axis represents the probability of identifying the disease, and the horizontal line is the prevalence of the disease. The predictiveness curve represents a spontaneous and graphical tool to compare the predictive power of any measured biomarker.

## Results

**Fig. 1:**
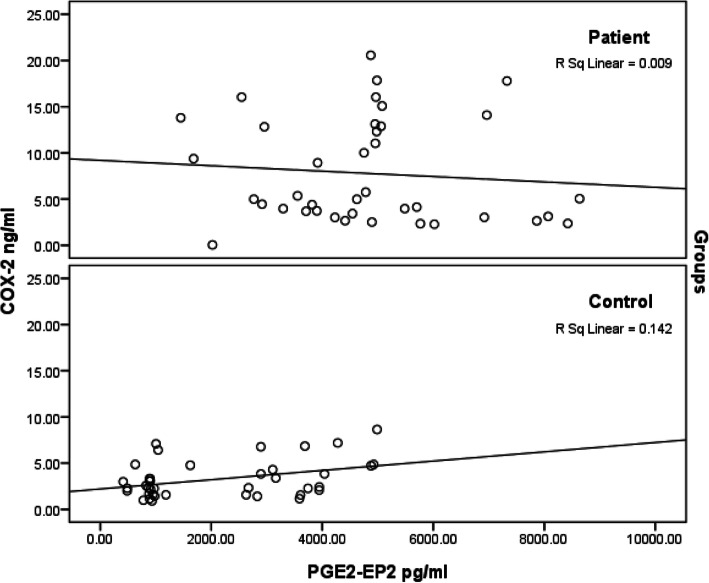
Pearson’s correlation of PGE2-EP2 and COX-2 patients and control healthy groups. Scatter plots depicted correlation of both variables with, the best fit negative correlation in a group of 40 patients (Up), and the best fit positive correlation curve in a group of 40 healthy control participants (Down)

Table 1 shows the primary data presented as the means ± SD and percent change for the three measured variables. Although plasma PGE-EP2 and COX-2 levels were significantly higher in individuals with ASD at 124 and 140%, respectively, compared to healthy controls (*P* < 0.001), α-Syn levels were decreased by 9.61% compared to healthy matched control participants, but the difference was not significant (*P* < 0.124). Figure 1 presents the positive correlations between COX-2 and PGE2-EP2 levels in the control group (*P*<0.016) and the non-significant negative correlation between both variables in the patient group (*P*< 0.576). Table 2 shows the results of the multiple regression analysis between the three measured variables, with PGE-EP2, COX-2, and α-Syn serving as dependent variables. The dependent variable PGE-EP2 was associated with COX-2 as a predictive value with an R^2^ value of 0.121, indicating that 12.1% of the increase in PGE-EP2 levels (Table 2) is due to the much higher PGE2 as product of COX-2 enzyme. The dependent variable COX-2 was substantially affected when both PEG-EP2 and α-Syn were used as predictor variables. Notably, 12.1% of its induction was related to PGE-EP2, and 16.7% was attributed to the combination of PGE-EP2 and α-Syn.

**Table 1:** Mean ± SD plasma levels of PGE2-EP2, COX-2, and α-Syn in patients with autism compared to healthy controls

Parameters	Groups	N	Min.	Max.	Mean ± SD	Percent change	*P* value
PGE2-EP2 (pg/ml)	Control	40	413.02	4987.98	2131.01 ± 1497.85	100.00	0.001
	Patient	40	1450.20	8635.27	4794.25 ± 1762.30	224.98	
COX-2 (ng/ml)	Control	40	0.91	8.64	3.28 ± 2.00	100.00	0.001
	Patient	40	0.05	20.57	7.89 ± 5.80	240.94	
α-Syn (pg/ml)	Control	40	8.20	31.54	17.19 ± 5.96	100.00	0.124
	Patient	40	8.51	40.66	15.54 ± 6.75	90.39	

Data were compared between groups using the Mann–Whitney test

**Table 2:** Multiple regression analysis using the stepwise method for PGE2-EP2, COX-2, and α-Syn as dependent variables

Dependent variable	Predictive Variable	Coefficient	S.E.	*P* value	Adjusted R^2^	95% CI	
						Lower	Upper
PGE2-EP2	COX-2	210.550	66.861	0.002	0.121	76.981	344.120
COX-2	PGE2-EP2	0.001	0.000	0.002	0.121	0.000	0.001
	PGE2-EP2 α-Syn	0.001	0.000	0.893	0.167	0.000	0.001
		0.143	0.067	0.003		0.009	0.277
α-Syn	COX-2	0.451	0.204	0.031	0.056	0.043	0.858

**Fig. 2:**
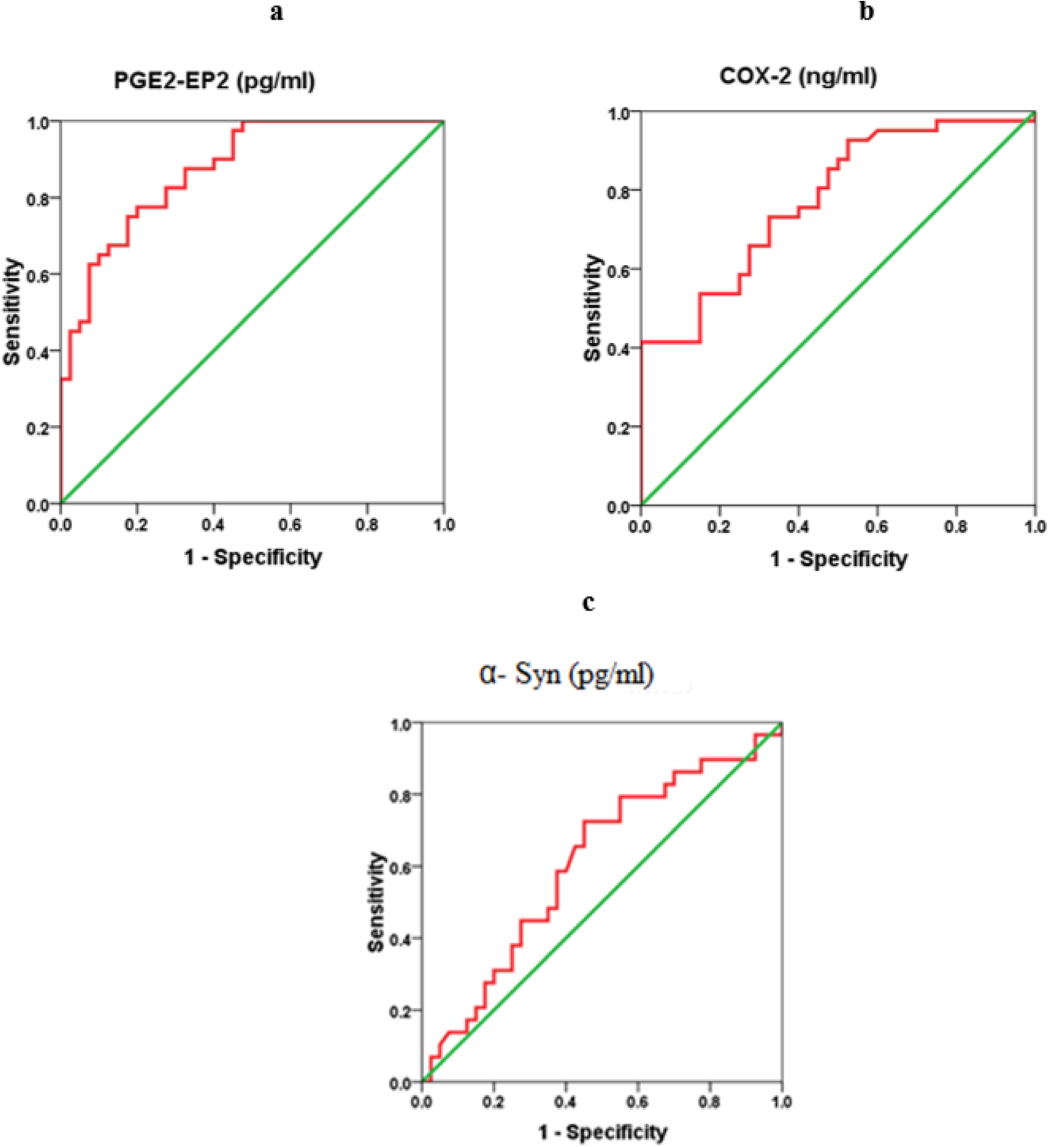
Receiver operating characteristic curve (ROC) for prediction of ASD based on the plasma levels of **a** PGE2-EP2, **b** COX-2, **c** α-Syn measured by ELISA, AUC (Area under the curve); PGE2-EP2: 0.875; COX-2: 0.776; α-Syn: 0.609

**Fig. 3:**
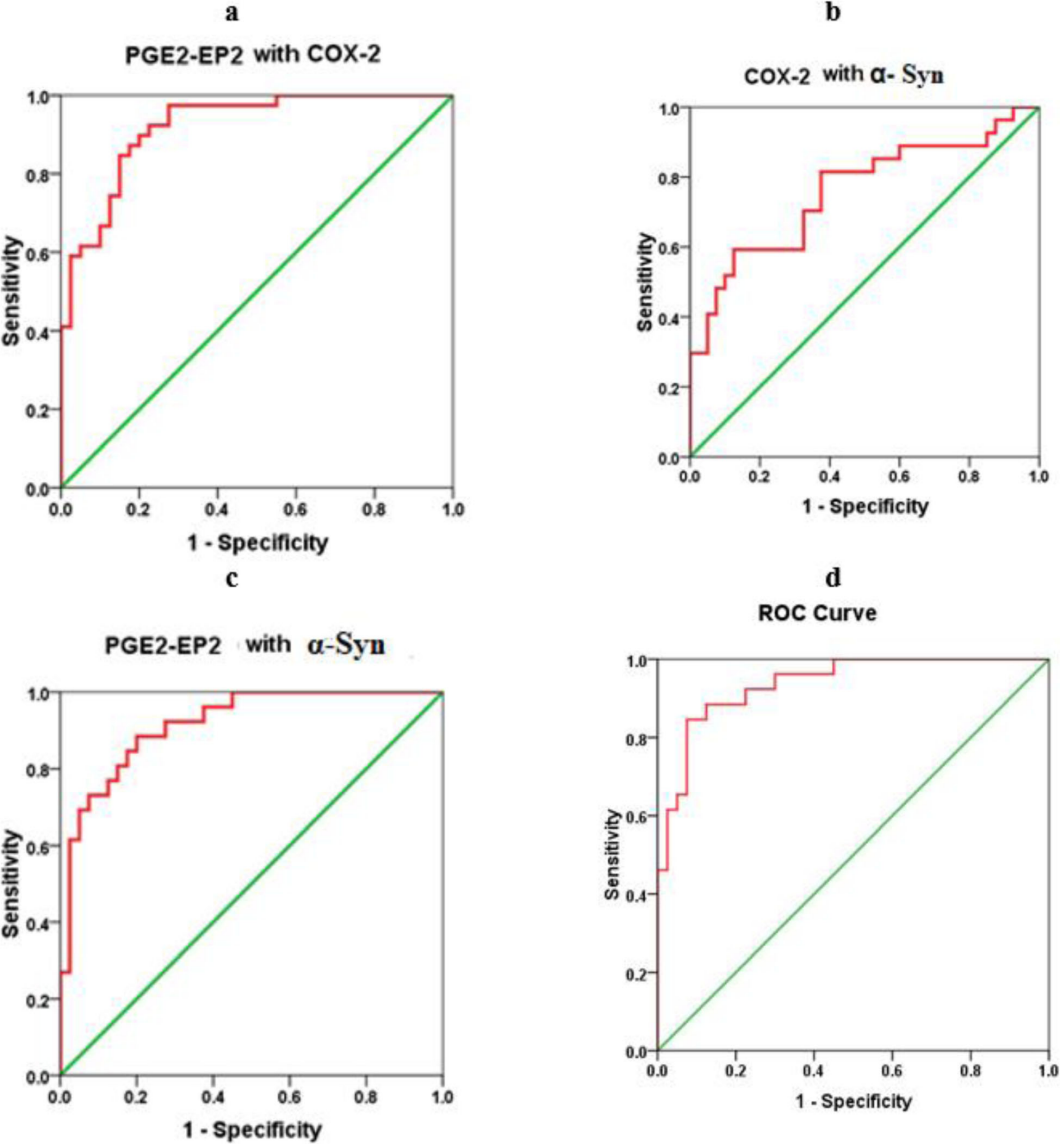
Receiver operating characteristic curves (ROC) for prediction of ASD based on the plasma levels of combining (**a**) PGE2-EP2 with COX-2, (**b**) COX-2 with α-Syn, (**c**) PGE2-EP2 with α-Syn, and (**d**) PGE2-EP2 with COX-2 and α-Syn in the patient group. AUC(Area under the curve); PGE2-EP2 with COX-2: 0.921; COX-2 with α-Syn: 0.891, PGE2-EP2 with α-Syn: 0.917; PGE2-EP2 with COX-2 and α-Syn: 0.938

**Fig. 4:**
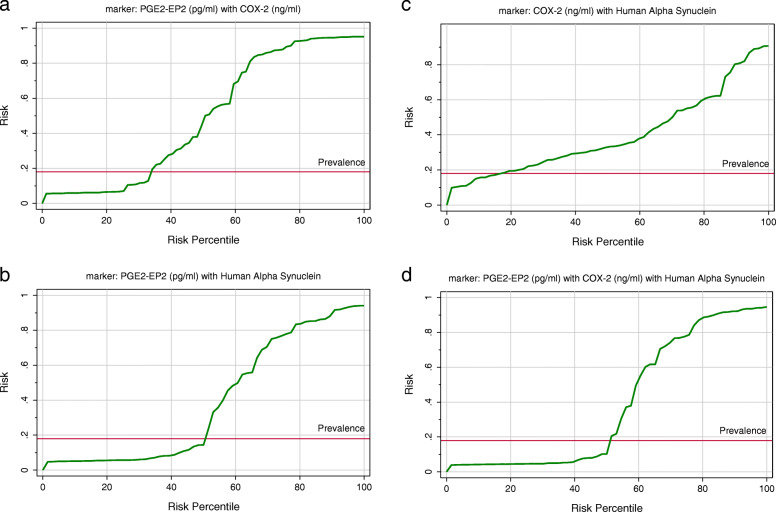
Predictiveness curve as graphical representation of the distribution of risk in the patient group by combining plasma (**a**) PGE2-EP2 with COX-2, (**b**) PGE2-EP2 with α-Syn, (**c**) COX-2 with α-Syn, and (**d**) PGE2-EP2 with COX-2 with α-Syn. The horizontal line of reference corresponds to the prevalence of the disease. The x axis represents the risk percentiles. The y axis represents the probability of identifying the disease

A ROC curve analysis was performed to evaluate the utility of these biomarkers in the early diagnosis of ASD. Figure 2 show the AUCs, cutoff values, specificity, and sensitivity of the three measured parameters independently. While PGE-EP2 alone recorded a satisfactory AUC of 0.875, both COX-2 and α-Syn independently showed fair and poor AUCs of 0.776 and 0.609, respectively. The low AUC of α-Syn was easily related to its nonsignificant decrease in patients with autism compared to controls (*P* < 0.124). As shown in Figs. 2 and 3, two models combining ROC curves were produced using logistic regression analyses as a tool. Figure 4 shows the predictiveness curves for the three measured variables. Of the three curves, only the PGE2-EP2 curve showed good predictive value (subjects at high and low risk were sufficiently far from the prevalence line). The two combining ROC models were effective at increasing the AUCs of the combined variables, as presented in Figs. 2 and 3, with a concomitant increase in the predictive values of the combined variables (Fig. 4). Despite the low AUC of α-Syn when presented independently (AUC = 0.609), when combined with PGE2-EP2, an excellent AUC of 0.917 was recorded.

## Discussion

In the present study, α-Syn levels were not significantly reduced in individuals with ASD, while significantly higher levels of both COX-2 and PGE-EP2 were observed in the plasma from patients with autism than in controls (Table 1). The high significant increase of PGE-EP2 transmembrane receptors in the plasma of ASD patients compared to healthy controls (Table 1) could be attributed to the most recently reported increase of ADAM10 and ADAM17 in the brain and intestine of ASD patients, as two sheddases that proteolytically cleave extracellular proteins [25]. Increase of PGE-EP2 transmembrane receptors in the plasma of ASD patients of course is concomitant with high rate of brain EP2 receptors deletion which from the theoretical point of view could decrease the pro-inflammatory effects of PGE2. Excitingly, the neuroprotection of PGE2-EP2 has been documented only recently, while a distinction among PGE2 receptors had not been made previously [31, 32]. Interestingly, Wu et al. and others reported that EP1 and EP3 receptor signaling has inflammatory affects in vitro and in vivo models of Cerebral hypoperfusion, or inadequate blood flow in the brain [33–36], as a well-known disorder in most ASD patients [37]. In contrast, EP2 receptor signaling protects neuronal cells under various conditions via the cAMP/PKA signaling pathway and controls microglial activation and function in vitro. These results reveal cell-specific variances in EP2 signaling among the four PGE receptors. Thus, the EP2 receptor may function inversely under acute and chronic neurological disorders. Based on this, the significant increase of PGE-EP2 plasma receptors reported in the current study (Table 1), and the concomitant EP2 brain receptor deletion could worsen brain injury, neuronal death, and neurobehavioral alterations in ASD patients [38].

Synucleinopathies are an accepted etiological mechanism of many neurological disorders [39, 40]. The mechanisms that trigger the unusual functions of α-Syn and how these mechanism modulate neurological pathogenesis remain poorly understood. Many studies have shown that α-Syn overexpression is related to mitochondrial dysfunction through the inhibition of complex I or complex IV activity [35, 36]. Recently, Lanoue et al. [41] suggested that neural development is substantially affected by synucleinopathies.

Although α-Syn is considered an intracellular protein, multiple reports establish the presence of α-Syn in biological fluids, such as cerebrospinal fluid (CSF) and blood plasma [19, 42–44]. In the present study, the non-significant but still considerable 9.61% lower levels of α-Syn in the plasma of children with ASD compared to controls were attributed to the disrupted blood–brain barrier (BBB) in patients with autism [17, 18, 45] and to the significant increase in levels of GM1 autoantibodies in children with autism compared to healthy controls as previously reported by Mostafa and AL-ayadhi [10]. Higher levels of autoantibodies against GM1, the most important ganglioside related to the clearance of α-synuclein from the brain, suggest that the reported lower level of α-Syn in plasma would be accompanied by higher levels in the brains of participants with ASD. Defects in the transport of α-Syn from the brain to blood might lead to an increased load of the inflammatory marker α-Syn within the brain of an individual with ASD. This suggestion is supported by the previous work of Sui et al. [16], who showed that α-Syn is transported across the BBB bidirectionally with a very rapid efflux rate (i.e., from the brain to the blood) of less than 2 minutes, and thus α-Syn the protein with the fastest efflux rate reported to date. Additionally, the remarkable but insignificant decrease in α-Syn levels reported in our study (Table 1) is supported by the work of Abou-Donia et al. [46] and Wang et al. [47]. In postmortem brain tissues from patients with α-synucleinopathy, α-Syn accumulation is associated with mitochondrial respiration defects, suggesting that mitochondrial dysfunction is a downstream effect of aggregated α-Syn. Based on this information, Fernández-Valle et al. [48] suggested that targeting the extracellular phase of α-Syn transmission is the most promising immunotherapy approach for Parkinson’s disease due its contribution to neuroinflammation.

The disruption of the electron transport chain (ETC) affects the reduction–oxidation (redox) potential of a cell and subsequent alters signaling pathways through the modulation of sirtuin proteins (SIRTs), contributing to cell survival/death [49]. Among mitochondrial SIRTs, SIRT3 is the best studied and was found to be responsible for the regulation of ETC protein complexes and the expression and activity of several antioxidant proteins, including superoxide dismutase (SOD2) and glutathione peroxidase (GPx). These proteins are vital for proper mitochondrial function and cell viability. Motyl et al. [49] showed that an increase in α-Syn levels in the brain increased the level of free radicals, decreased the mitochondrial membrane potential, decreased cell viability, and activated cell death through its effect on SIRT3 as a mitochondrial protein. Therefore, we suggest that the significant decrease in α-Syn levels observed in our study occurs concomitant with mitochondrial respiration defects, suggesting that mitochondrial dysfunction is a downstream effect of aggregated α-Syn in the brain.

Fig. 2 shows the significant positive correlations between COX-2 and PGE2-EP2 levels in the control group (*P*<0.016) and the non-significant negative correlation between both variables in the patient group (*P*< 0.576). An exciting approach to explain this variation is based on the fact that activation of the EP2 receptor is neuroprotective under physiological or mild injury conditions as EP2 receptor deletion worsened brain injury, neuronal death, and neurobehavioral insufficiencies, while EP2 receptor activation by a highly selective agonist reversed these effects [36]. Interestingly, in acute glutamate excitotoxicity models and patients with ASD, inhibition of protein kinase A (PKA), a cyclic AMP-dependent protein kinase, reverses the protective effect of EP2 signaling, indicating that neuronal EP2-mediated protection depends on cAMP signaling [50]. This explanation is acceptable, as ASD is at least partially associated with decreased PKA-mediated phosphorylation of proteins and abnormalities in cellular signaling [51, 52].

As shown in Table 2, the multiple regression analysis indicated a substantial effect of both PGE2-EP2 and α-Syn as predictors or independent variables on the dependent variable Cox-2 (adjusted R^2^ of 0.167).

The increased diagnostic value of the combination of α-Syn and COX-2 (AUC of 0.785) shown in Table 4 and Fig. 3 compared to the independent diagnostic values of both proteins alone (AUC of 0.609 and 0.776, respectively) (Fig. 2) was confirmed to improve predictiveness curves (Fig. 4) compared to the independent variables. This result might be attributed the effect of increased COX-2 expression on inducing oxidative protein modification and α-Syn accumulation in dopaminergic cells, concomitant with much lower plasma levels [53, 54].

Moreover, the increased diagnostic value of the combination of α-Syn and PGE2-EP2 (AUC of 0.917) (Fig. 3) compared to the independent diagnostic value of both parameters alone (AUC of 0.609 and 0.875, respectively), as shown in Fig. 2, is explained by the relationship of the markers. Microglial phagocytosis of α-Syn aggregates occurs at a much higher rate in the brains of PGE2-EP2 knockout mice (EP2^−/−^). Because levels of PGE2, a product derived from AA by COX and specific synthases, are also significantly elevated in the plasma and CSF of individuals with ASD, the finding that significantly lower levels of α-Syn correlated with a significant increase in PGE2-EP2 levels in the present study was interesting. This finding supports the work of Jin et al. showing that signaling through the EP2 receptor induces α-synuclein aggregate-mediated neurotoxicity in the brains of rodent models of Parkinson’s disease [27]. This relationship is clearly shown in Fig. 3d, in which the AUC of the three combined variables showed the highest value either compared to the model of ROC curves combining 2 parameters (Fig. 3a, b, and c) or the independent, uncombined ROC curves (Fig. 2). Again, this result was clearly observed in the perfect predictiveness curves of the three combined variables (Fig. 4).

Kadak et al. [55] recorded that the significantly lower levels of α-Syn and tau in the serum of patients with ASD support their involvement in synaptic abnormalities as etiological mechanisms of ASD. The insignificant decrease in plasmatic α-Syn aggregate reported in the current study, which contradicts the highly significant decrease reported by Kadak et al. [55], could be attributed to the differences in the population and the demographic characteristics of the participants. Here, we will explain the remarkable increase in the diagnostic value of the combination of the three markers (α-Syn, COX-2, and PGE2-EP2) through their relationship to glutamate excitotoxicity as a major synaptic abnormality repeatedly reported in individuals with ASD. According to Dos-Santos-Pereira et al. [56], the activation of microglial cells by α-Syn aggregates may result in an excitotoxic accumulation of extracellular glutamate. COX-2 plays a key role in altering excitatory neurocircuitry [57–59]. COX-2 expression is upregulated by N-methyl-D-aspartate (NMDA) receptor activation during both balanced glutamatergic excitation and excitotoxicity, suggesting that shifts in glutamatergic activity in individuals with ASD affect COX-2 signaling and are directly related to the loss of synaptic plasticity-induced neural damage ultimately leading to complex social and cognitive dysfunction as features of ASD [57–61]. Interestingly, COX-2 inhibition with a concomitant attenuation of PGE2/EP2 in rat cortexes is accompanied by significant inhibition of glutamate release and avoidance of glutamate excitotoxicity which have been implicated in epilepsy as co-morbidity in individuals with autism [62, 63]. This does not contradict the suggested deleterious effects of PGE2/EP2 shedding from the brain to plasma in ASD patients, as reported in the current study. Among the four subtypes of EP receptors, EP1 has the lowest affinity for PGE2 [64]. Based on this, deletion of EP2 receptors from the brain could be concomitant with activation of EP1 receptors due to the higher availability of PGE2 as a ligand. It is well documented that the EP1 receptor contributes to excitotoxicity and focal ischemic brain damage through PGE2-induced intracellular calcium mobilization [65–67]. This explanation can find support in the study of Gendron et al. [68] which reported that in the presence of a COX-2 inhibitor, excitotoxicity induced neuronal death could be still elicited with an EP1 receptor agonist, suggesting that among the four EP receptors, there were protective (e.g. EP2) as well as toxic (e.g. EP1) receptors [23]. The ultimate output of PGE2 signaling is determined and greatly affected by the expression of each EP receptor and the strength of each EP signal [69].

Interestingly, a most recent randomized, parallel-group pilot clinical study demonstrates the improvement of ASD behavior assessed with Childhood autism rating scale (CARS) and α-Syn levels in ASD patients after consumption of a beta-glucan prebiotic, which showed the relationship between α-Syn protein aggregates as an indicator of ASD severity [70] and altered gut microbiota as another phenotype of this disorder [71].

**Fig. 5:**
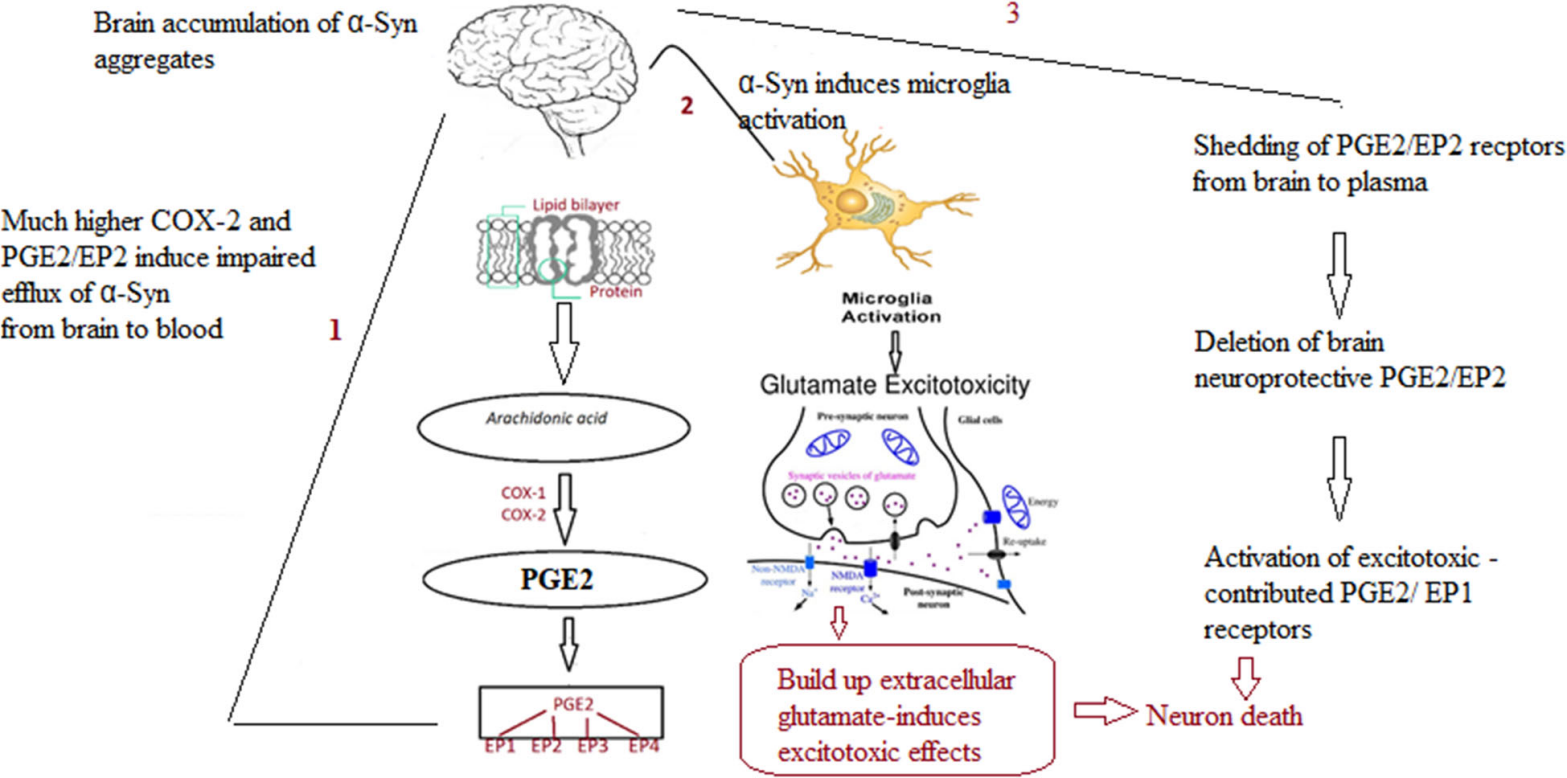
Illustration of the relationship between α-Syn, COX-2, and PGE2/EP2 in inducing glutamate excitotoxicity and PGE2/EP2 shedding as etiological mechanisms of ASD

The suggested relationship between α-Syn, COX-2, and PGE2/EP2 in inducing glutamate excitotoxicity and mitochondrial dysfunction as two etiological mechanisms in ASD is illustrated in Fig. 5.

### Comparisons with other studies and what does the current work add to the existing knowledge

Despite the reported dozens of neurophysiological and biochemical markers published during the last 10 years in ASD children, however, no accurate biomarker has emerged, and the etiological mechanisms of ASD are still not fully understood to be targeted as intervention.

### Study strengths

The application of combining ROC could increase the diagnostic value of each independent biomarker and help to understand the relationship and signaling pathways between the combined panels of markers as a tool for advancing our understanding of the etiological mechanisms of ASD.

### Study limitations

The main limitation of the present study is the absence of subgrouping according to the severity of the disorder among the ASD participants. In addition, the design of ROC curves by comparing the biomarkers in ASD against neurotypical healthy controls rather than against other neurological or neurodevelopmental disorders can be considered as another limitation.

## Conclusions

The remarkable increase in the predictive diagnostic value of combining α-Syn, COX-2, and PGE2/EP2 as three biomarkers of ASD, together with their role in mitochondrial dysfunction and glutamate excitotoxicity as two confirmed etiological mechanisms of ASD, suggests that the variables might serve as useful diagnostic markers for this disorder that might help the early intervention.

## Data Availability

Data will be available upon request.

## Abbreviations

ASD: Autism spectrum disorder
α-Syn: Alpha-synuclein
ART: Autism Research & Treatment
ADI-R: Autism Diagnostic Interview-Revised
ADOS: Autism Diagnostic Observation Schedule
COX: Cyclooxygenase
COX 2: Cyclooxygenase-2
CNS: Central nervous system
CSF: Cerebrospinal fluid
3DI: Developmental, Dimensional, and Diagnostic Interview
ELISA: Enzyme-linked immunosorbent assay
ETC: Electron transport chain
GPx: Glutathione peroxidase
GPCRs: G protein-coupled receptors
NMDA: N-methyl-D-aspartate
ORs: Odds ratios
PGE2: Prostaglandins-EP2
ROC: Receiver operating characteristic
SOD: Superoxide dismutase
