# Correction to: Alpha-Synuclein, cyclooxygenase-2 and prostaglandins-EP2 receptors as neuroinflammatory biomarkers of autism spectrum disorders: Use of combined ROC curves to increase their diagnostic values

**DOI:** 10.1186/s12944-021-01598-3

**Published:** 2021-11-28

**Authors:** Afaf El-Ansary, Manan Alhakbany, Abeer Aldbass, Hanan Qasem, Sarah Al-Mazidi, Ramesa Shafi Bhat, Laila Al-Ayadhi

**Affiliations:** 1 Central Laboratory, Female Center for Medical Studies and Scientific Section, King Saud University, P. O Box 22452, KSA Riyadh, 11495 Saudi Arabia; 2 Autism Research and Treatment Center, Riyadh, Saudi Arabia; 3 Department of Physiology, Faculty of Medicine, King Saud University, Riyadh, Saudi Arabia; 4 Biochemistry Department, College of Science, King Saud University, Riyadh, Saudi Arabia; 5 Department of Physiology, College of Medicine, Al-Imam Mohammed Bin Saud Islamic University, Riyadh, Saudi Arabia

**Correction to: Lipids Health Dis 20, 155 (2021).**

**https://doi.org/10.1186/s12944-021-01578-7**

Following publication of the original article [1], the authors noticed that the funding statement is incorrect and needs amendment to match the acknowledgement. Please see below correct funding statement.
